# Ultrafast time-resolved electron diffraction revealing the nonthermal dynamics of near-UV photoexcitation-induced amorphization in Ge_2_Sb_2_Te_5_

**DOI:** 10.1038/srep13530

**Published:** 2015-08-28

**Authors:** Masaki Hada, Wataru Oba, Masashi Kuwahara, Ikufumi Katayama, Toshiharu Saiki, Jun Takeda, Kazutaka G. Nakamura

**Affiliations:** 1 Materials and Structures Laboratory, Tokyo Institute of Technology, Yokohama 226-8503, Japan; 2 PRESTO, Japan Science and Technology Agency, Kawaguchi 332-0012, Japan; 3 Department of Physics, Graduate School of Engineering, Yokohama National University, Yokohama 240-8501, Japan; 4 Electronics and Photonics Research Institute, National Institute of Advanced Industrial Science and Technology, Tsukuba 305-8562, Japan; 5 Graduate School of Science and Technology, Keio University, Yokohama 223-8522, Japan; 6 CREST, Japan Science and Technology Agency, Kawaguchi 332-0012, Japan

## Abstract

Because of their robust switching capability, chalcogenide glass materials have been used for a wide range of applications, including optical storages devices. These phase transitions are achieved by laser irradiation via thermal processes. Recent studies have suggested the potential of nonthermal phase transitions in the chalcogenide glass material Ge_2_Sb_2_Te_5_ triggered by ultrashort optical pulses; however, a detailed understanding of the amorphization and damage mechanisms governed by nonthermal processes is still lacking. Here we performed ultrafast time-resolved electron diffraction and single-shot optical pump-probe measurements followed by femtosecond near-ultraviolet pulse irradiation to study the structural dynamics of polycrystalline Ge_2_Sb_2_Te_5_. The experimental results present a nonthermal crystal-to-amorphous phase transition of Ge_2_Sb_2_Te_5_ initiated by the displacements of Ge atoms. Above the fluence threshold, we found that the permanent amorphization caused by multi-displacement effects is accompanied by a partial hexagonal crystallization.

One of the major applications for laser-induced solid-to-solid phase transitions is nonvolatile optical storage devices, *i.e.*, phase-change memories: such as compact discs, digital versatile discs and Blu-ray discs^1^,^2^,^3^,^4^. Among optical media materials, chalcogenide glasses have several advantages, including a high writing speed (~10 ns bit^–1^), direct overwriting capability (10^6^ cycles), and resistance to climate and natural light^5^,^6^,^7^,^8^. The phase transitions in this class of materials are usually induced by nanosecond or continuous-wave lasers via thermal processes involving melting and quenching of the crystal-to-amorphous transition with subsequent annealing to recover the original crystalline phase. As the Internet requires faster and denser devices, the key challenges in phase-change memories, such as improving memory access, encoding speed, and understanding phase transition and damage mechanisms, should be addressed.

Recently, a nonthermal phase transition of the Ge_2_Sb_2_Te_5_ (GST) poly-crystal or the GeTe–Sb_2_Te_3_ superlattice by an ultrashort pulsed laser has been proposed, which may realise ultrafast memory encoding^9^,^10^,^11^. The dynamics of this ultrafast phase transition in GST has been studied using time-resolved spectroscopy^12^,^13^,^14^,^15^, and x-ray and electron diffraction^16^,^17^. Time-resolved optical studies have demonstrated that the optical index of GST changes from opaque to transparent within 1 ps after near-infrared (IR) photoexcitation, with a clear threshold in the fluence of the incident laser for permanent amorphization^12^,^13^. Time-resolved x-ray spectroscopy has suggested that the amorphization of GST is initiated by the displacement of Ge atoms from an octahedral to a tetrahedral arrangement^15^. The displacements of Ge atoms, referred to as local amorphization^9^ by A.V. Kolobov, *et al.*, is distinct from highly disordered amorphization. A germanium atom travels approximately 0.29 nm from an octagonal to a tetragonal site in this process. Acoustic phonon propagation and thermally limited amorphization have been observed by time-resolved x-ray and electron diffraction experiments with near-IR photoexcitation^16^,^17^. In essence, the thermal nature of the laser-induced amorphization of GST deduced from crystallographic methods conflicts with the results obtained from time-resolved x-ray and optical spectroscopy^9^,^15^. These observations are in line with the energy difference of ~1 eV between the incident photon (1.55 eV) and the bandgap in GST (0.5–0.6 eV)^18^. The nonthermal signals in time-resolved crystallography may be buried in the thermal contribution caused by this energy difference. As suggested from the electronic band structure^19^ and in contrast to the above observation, visible to near-UV photoexcitation brings the system to a different excited state that can also induce amorphization along the edge of the potential energy surface. Besides, the absorption spectrum reaches a maximum in visible range (around 2.6 eV)^20^. Here, the amorphization dynamics in GST induced by visible to near-ultraviolet (UV) photoexcitation should fully exploit the nonthermal phenomena in optical media materials.

In this report, we investigate the mechanism of the crystal-to-amorphous phase transition process followed by near-UV (400 nm) femtosecond laser excitation on 20-nm-thick polycrystalline GST films using time-resolved electron diffraction (Tr-ED) measurements and single-shot time-resolved optical spectroscopy. The advantage of Tr-ED measurements lies in the fact that the penetration depth of the near-UV pump light (~20 nm) for the phase transition matches the sample thickness required for the diffraction measurement^21^,^22^,^23^. Our experimental results on the intensity changes in five diffraction rings present local amorphization in GST on a timescale of 10–20 ps *i.e.*, the displacement of Ge atoms from an octahedral to a tetrahedral arrangement^9^. Below the critical laser fluence, this local amorphization reverts to the initial state by local annealing. We also found that the local amorphization evolves to more complicated permanent amorphization via multi-displacement effects above this fluence threshold when more than 25% local amorphization occurs in the system. Above this fluence threshold, the permanent amorphization is accompanied by partial hexagonal recrystallization, which may be one of the damage processes in optical storage.

## Results

### Time-resolved optical transmission of GST

**Figure 1:**
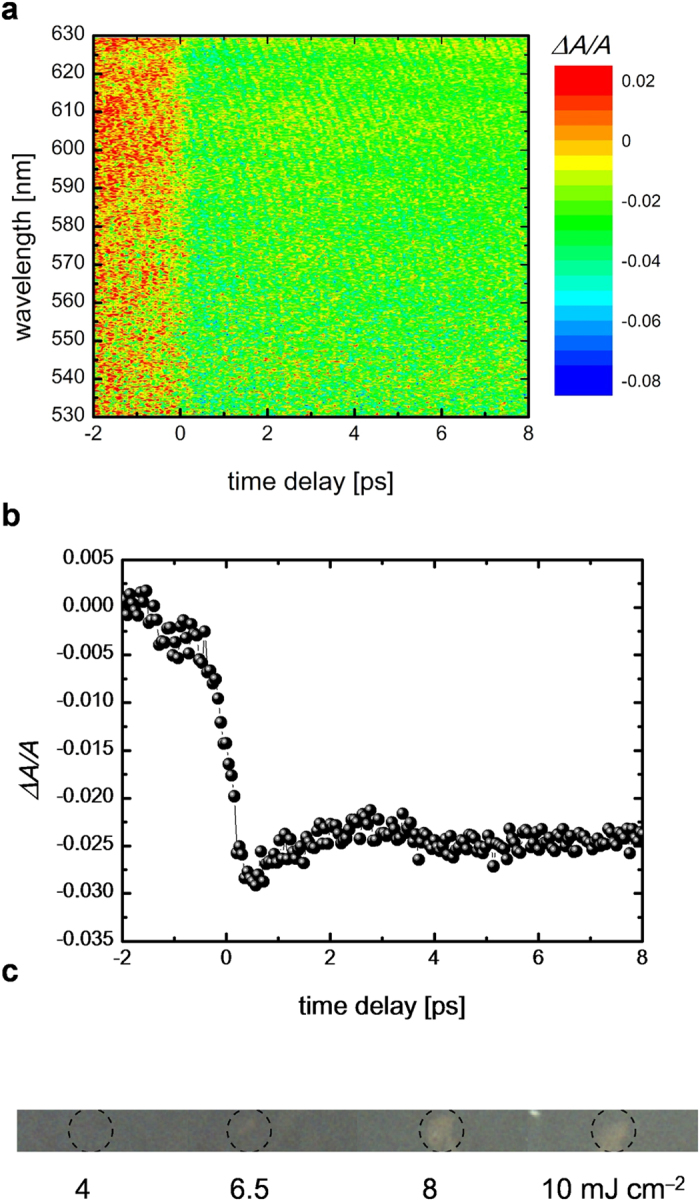
Near-UV pump and visible probe spectroscopy. (**a**) Two-dimensional plot of the time-resolved differential absorbance (Δ*A/A*) as a function of probe photon wavelength. (**b**) Averaged Δ*A/A* for wavelengths of 530–630 nm as a function of time delay. (**c**) Photographs after single-shot near-UV (400 nm) photoexcitation at various incident fluences (4, 6.5, 8 and 10 mJ cm^–2^). Legends are shown as insets in the figure. The permanent amorphization fluence threshold was determined to be approximately 8 mJ cm^–2^.

Here we present results from single-shot time-resolved transmission spectroscopy of GST: the “on” and “off” states of the optical media materials are recognized by a change in optical index. Figure 1a,b present the near-UV (400 nm) pump and visible probe transmission spectra as a function of probe photon wavelength in the range of 530 nm to 630 nm. The changes in the optical index of GST were identical upon photoexcited by 400-nm or 800-nm light^12^,^14^ suggesting that the opaque rock-salt GST is electronically stabilized into transparent amorphized-GST within 1 ps. The threshold for the permanent amorphization is determined as a laser incident fluence (*F*) of 8 mJ cm^–2^ (Fig. 1c) which is smaller than that obtained in the case of near-IR photoexcitation (>10 mJ cm^–2^).

### Tr-ED measurements of GST at the photoexcitation below the fluence threshold

**Figure 2:**
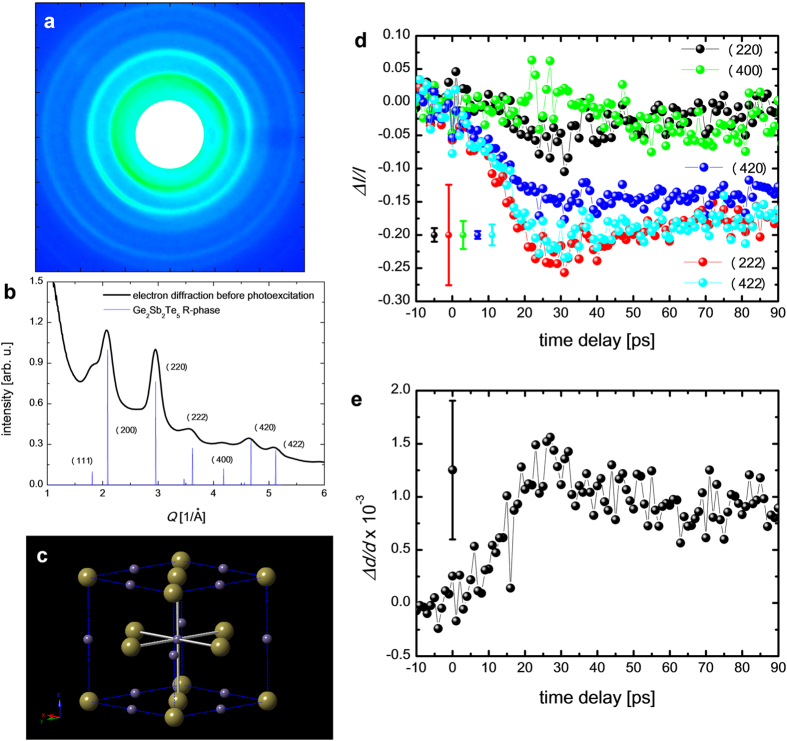
Tr-ED for photoexcitation below the fluence threshold. (**a**) Electron diffraction pattern before photoexcitation. (**b**) Radial average compared with simulated diffraction of R-phase GST. (**c**) Crystal unit cells of R-phase GST. The yellow spheres indicate tellurium atoms, and the purple spheres indicate germanium atoms (40%), antimony atoms (40%) and vacancies (20%). (**d**) Time evolution of the electron diffraction intensity from the (200), (222), (400), (420) and (422) planes. Legends and typical errors for each diffraction intensity are shown as insets in the figure. (**e**) Time evolution of average lattice expansions obtained from the (200), (222), (400), (420) and (422) electron diffraction rings. Inset vertical error bars indicate the standard deviation from the mean values of the change in electron diffraction intensity and in *Q* value.

The electron diffraction pattern (Fig. 2a) from the undisturbed 20-nm-thick GST represents a polycrystalline rock-salt structure with Miller-indices assigned to the peaks as shown in Fig. 2b. GST in optical media or thin film annealed at temperature of 150–200 °C exhibits a metastable rock-salt structure^24^ (F*m*3*m*, R-phase) as shown in Fig. 2c. In the R-phase, the Te atoms occupy the sites on one face-centre-cubic lattice, with Ge (40%) atoms, Sb (40%) atoms and vacancies (20%) randomly forming the other face-centre-cubic lattice. This static electron diffraction pattern concurs with a previous report^17^. The diffraction rings from the (111) and (200) planes overlapped; therefore, we analysed the peak intensities and positions (*Q*-values: *Q* = 2*π*/*d*, where *d* is lattice spacing) from the (220), (222), (400), (420) and (422) planes.

The time evolution of the intensity of these diffraction rings followed by irradiation with 400-nm light with an *F* of 5 mJ cm^–2^, is shown in Fig. 2d. Below an *F* value of apploximately 8 mJ cm^–2^, the photoexcitation phenomenon is in a repetitive regime. The changes in diffraction intensities for the (220) and (400) planes with the photoexcitation are negligibly small. However, those for the (222), (420) and (422) planes decreased by approximately 15% with the photoexcitation in approximately 10–20 ps following photoexcitation. This period corresponds to the reported amorphization period studied in previously molecular dynamics calculation and time-resolved x-ray and electron diffraction experiments^15^,^17^,^25^. All the *Q*-values have negative shifts suggesting that the rock-salt lattice of GST expands after photoexcitation (Fig. 2e). By calculating the lattice expansion with a linear expansion coefficient^26^ (1.33 ± 0.14 × 10^–5^ K^–1^) in amorphous phase GST, the increase in the lattice temperature is approximately 70–100 K.

**Figure 3:**
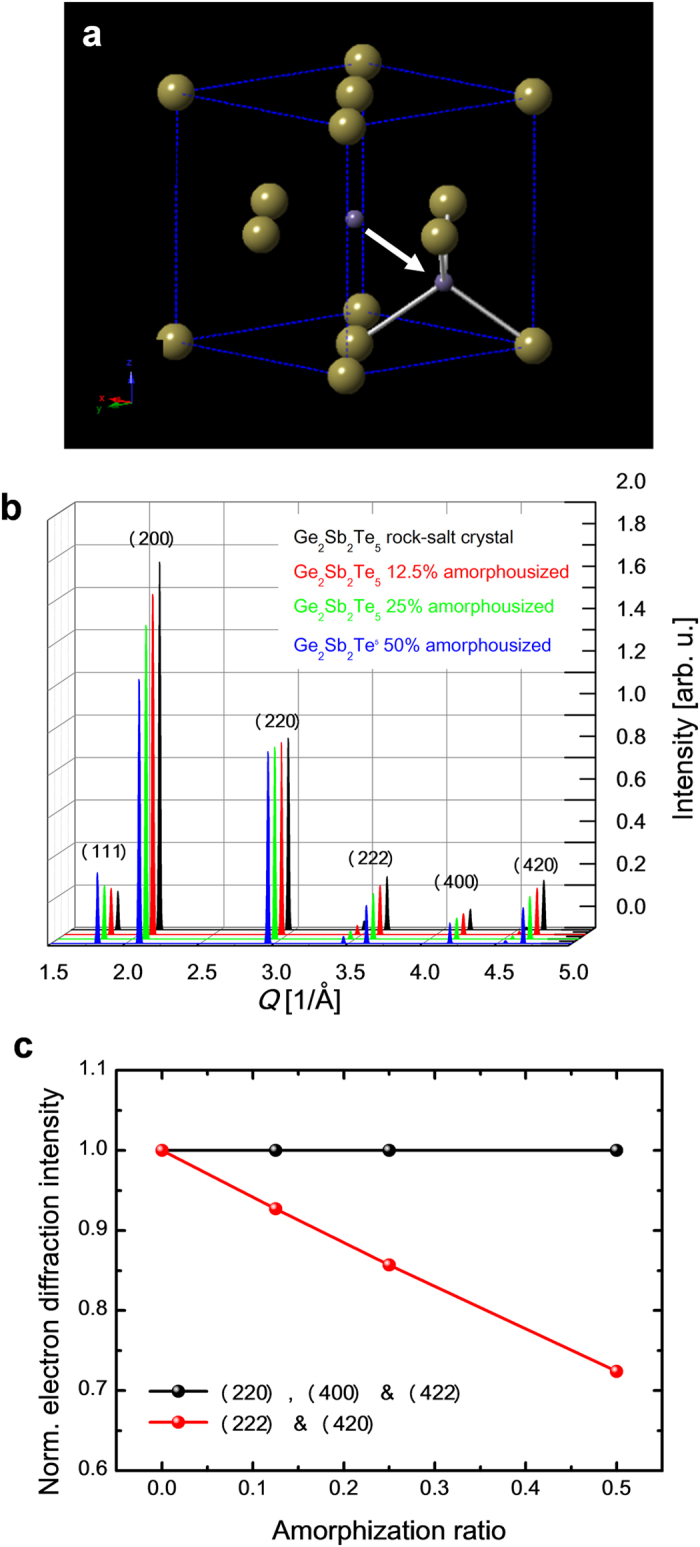
Effect of amorphization on electron diffraction intensity. (**a**) Local amorphization with disordering of Ge atoms from an octahedral site to a tetrahedral site in R-phase GST. (**b**) Calculated electron diffraction intensity at a local amorphous ratio of 0% (perfect GST rock-salt crystal), 12.5%, 25%, and 50%. (**c**) Calculated relative electron intensities from the (220), (222), (400), (420) and (422) planes compared with those from perfect R-phase GST crystal as a function of the local amorphous ratio.

The decrease observed in the electron diffraction intensities of a few peaks drop upon the photoexcitation with a constant crystal symmetry suggests that the amorphization is due to the displacements of specific atoms in a GST unit cell, without a change in the long-range structural periodicity. The thermal amorphization in GST has been reported to be rather complicated creating ring-like structures^27^,^28^; however, our finding indicates simple local amorphization as a displacement of Ge atoms from an octahedral to a tetrahedral arrangement^9^ as shown in Fig. 3a. These results for near-UV photoexcitation differ from previous findings obtained for time-resolved x-ray and electron diffraction measurements employing near-IR photoexcitation.

Figure 3b,c show the calculated electron diffraction intensity of perfect R-phase GST and R-phase GST with octagonal Ge vacancies and tetrahedral Ge interstitial sites. The local amorphous ratio (*R*_*a*_) indicates the proportion of Ge atoms in tetrahedral sites with respect to the total number of Ge atoms. There are four octahedral sites in a unit cell of GST, and the Ge atoms occupy 1.6 sites. The local amorphous ratio is expressed by the average number of Ge atoms transiting to the tetrahedral interstitials (*N*_*I*_) in a unit cell as *R*_*a*_=*N*_*I*_/1.6. The electron diffraction intensities from the (220), (400) and (422) planes are independent of the local amorphous ratio. On the other hand, the relative electron diffraction intensities from (222) and (420) planes decrease linearly as the local amorphization ratio increases (Fig. 3c). To estimate the ratio of amorphization, the decreases in electron diffraction intensities (Δ*I*/*I*) with increasing temperature (Δ*T*) was also calculated. According to the kinematic theory of diffraction Δ*I*/*I* is expressed as^24^,^29^,^30^:


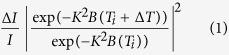


where, *K*, *B*(*T*), and *T*_*i*_ are scattering vector, the *B*-factor (Debye-Waller factor)^31^ and the initial temperature of the sample, respectively. With a temperature increase of 70–100 K, the decrease in diffraction intensities of the (220), (222), (400), (420) and (422) planes are calculated to be 3.4 ± 0.4%, 5.0 ± 0.7%, 6.6 ± 0.9%, 8.1 ± 1.2% and 9.8 ± 1.3%, respectively. The diffraction intensity drops in the (222) and (420) planes shown in Fig. 2c should be due to the combination of thermal and local amorphization effects. The contribution of local amorphization to the decrease in electron diffraction intensity in the (222) and (420) planes is obtained by a simple subtraction as 10 ± 7% and 7 ± 2%, respectively. The local amorphization ratio at this photoexcitation level (*F*: 5 mJ cm^–2^) is obtained as 10–16% from Fig. 3c.

### Tr-ED measurements on GST for photoexcitation above the fluence threshold

**Figure 4:**
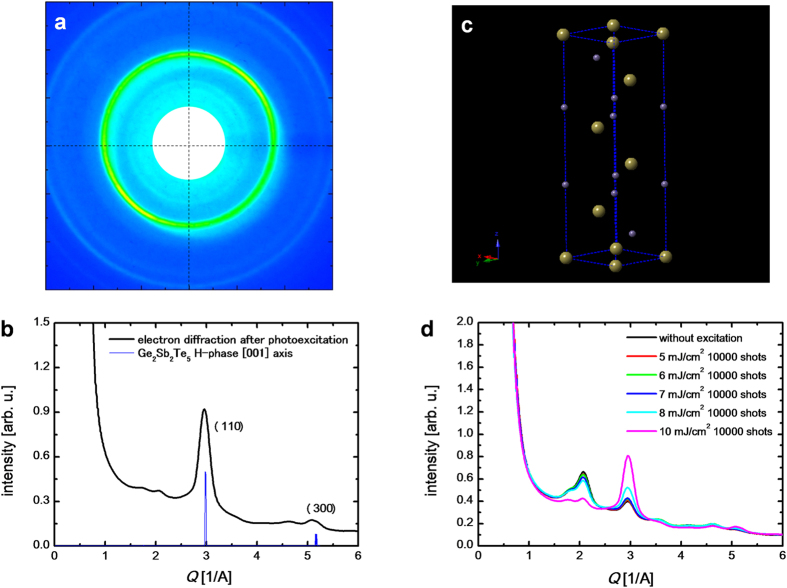
Electron diffraction for photoexcitation above the fluence threshold. (**a**) Electron diffraction pattern after irradiation with 5 × 10^4^ shots of 400-nm light at a fluence of 10 mJ cm^–2^. (**b**) Radial average compared with the simulated diffraction intensity of GST in the H-phase perpendicular to the [001] axis. (**c**) Crystal unit cells of GST in the H-phase. (**d**) Intensities of electron diffraction after irradiation with 1 × 10^4^ shots of 400-nm light at an incident fluence range of 5 to 10 mJ cm^–2^. Legends are shown as insets in the figure.

The electron diffraction patterns after photoexcitation with higher incident fluences show clear differences compared to that of the undisturbed R-phase GST structure (Fig. 4a). As shown in Fig. 4b, the diffraction ring at a *Q*-value of 2.95 has a slight positive shift, and a new peak at a *Q*-value of 5.15 appears with the other peaks from rock-salt GST decreasing. This structure is assigned to the polycrystalline hexagonal phase (H-phase) of GST oriented on the [001] axis perpendicular to the normal of the substrate because the peaks at the *Q*-value of 2.95 and 5.15 corresponds to two unique diffraction peaks from the (110) and (300) planes of the H-phase perpendicular to the *c*-axis, respectively. GST in a stable hexagonal^32^,^33^ (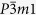) crystal structure (Fig. 4c) is obtained via higher temperature annealing processes (350–500 °C); therefore, photoexcitation with a higher incident fluence should induce the rocksalt-to-hexagonal phase transition of GST through partially thermalization or melting. Thermal segregation of tellurium in GST has been reported in the literature^34^; however, this structure differs from that of segregated tellurium. H-phase recrystallization has never been observed by optical studies because the optical refractive index between the R-phase and the H-phase is smaller than that between the R-phase and the amorphous phase^35^,^36^, and because a small amount of H-phase is created by the single pulse irradiation.

**Figure 5:**
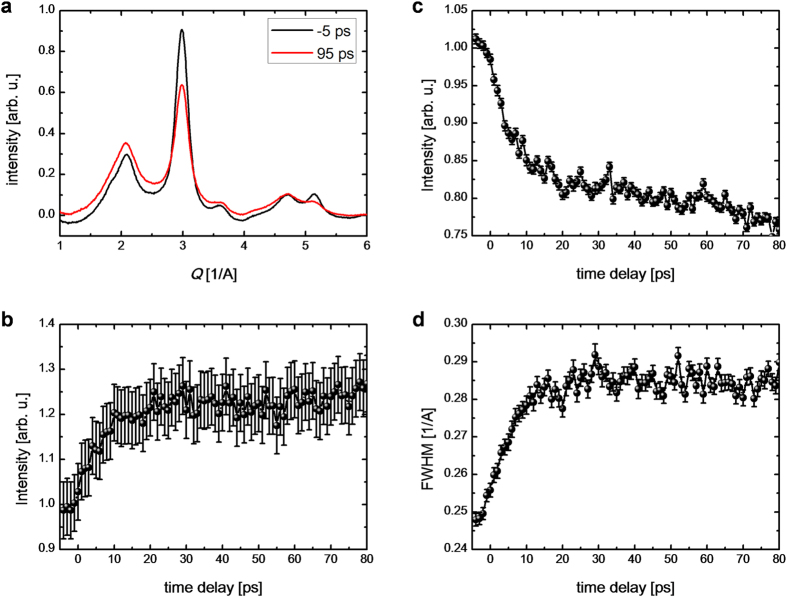
Tr-ED for photoexcitation above the fluence threshold. (**a**) Tr-ED intensities before (–5 ps) and after (95 ps) photoexcitation. The fluence of the incident laser was 10 mJ/cm^2^. Because of the high fluence of the incident laser, the repetition rate of the laser for this time-resolved experiment was limited to 100 Hz. (**b**) Time evolution of the base level intensity. (**c**) Time evolution of the electron diffraction intensity from (110) H-phase GST. (**d**) Time evolution of full-width-half maximum (FWHM) of (110) H-phase GST. Inset vertical error bars indicate the standard deviation from the mean values of the change in electron diffraction intensity and in FWHM.

As the fluence of the incident laser increases, the photoexcitation phenomenon enters the irreversible regime. The fluence threshold of the rocksalt-to-hexagonal phase transition is found to be approximately 8 mJ cm^–2^ (Fig. 4d), which corresponds to that of permanent amorphization obtained by the time-resolved optical measurements. The optical pump-probe results suggest that the GST film irradiated with higher fluence photoexcitation is in a permanent amorphous phase. In general, it is quite challenging to observe the amorphous contribution using crystallographic methods when a sample is composed of both amorphous and crystalline structures. Despite being in the irreversible regime, we can still follow the H-phase-crystal-to-amorphous phase transition with repetitive time-resolved electron diffraction. Figure 5a presents the profiles of the electron diffraction before and after the photoexcitation. The time evolution of intensity of base level is shown in Fig. 5b and the time evolution of intensity and full-width-half maximum of the (110) H-phase GST diffraction peak are shown in Fig. 5c,d. The increase in the base level, decrease in the intensity of the peak and peak broadening provide clear evidence of strong disordering in the system (melting or amorphization) by the higher fluence photoexcitation^37^,^38^, which is not observed for photoexcitation below the fluence threshold. These changes occur on the timescale of around 10 ps. Thus, the GST film irradiated with a higher fluence photoexcitation contains both permanent amorphous phase and H-phase GST. The permanent amorphous phase obtained with a higher fluence photoexcitation has a more complicated amorphous structure than the local amorphization induced by a lower fluence photoexcitation (below the threshold).

## Discussion

Unlike thermal mechanisms, laser-induced nonthermal processes are determined by the number of photons absorbed in the system. The number of Ge atoms per unit volume is 7.4 × 10^21^ cm^–3^, as a unit cell (2.2 × 10^–22^ cm^3^) contains 1.6 Ge atoms (4 sites with 0.4 occupations). Assuming a homogenous excitation in the 20-nm-thick-GST film, the number of absorbed photons in a unit volume at an *F* value of 5 mJ cm^–2^ is calculated to be 2.0 × 10^21^ cm^–3^ with a reflectivity of 60% and a single photon energy of 3.1 eV. The discussion above suggests that 27% of Ge atoms in the system are excited. The local amorphization ratio (10–16%) induced by photoexcitation at an *F* value of 5 mJ cm^–2^ is on the same order of magnitude as the ratio of photoexcited Ge atoms (27%), reflecting that the amorphization process of GST is based on the very nature of the laser-induced nonthermal process. As the ratio of local amorphization increases linearly with the fluence of the incident laser, *i.e.*, the number of absorbed photons in the system, the threshold of permanent amorphization corresponds to the displacement of 25% of the Ge atoms from octahedral to tetrahedral sites, which is close to the maximum limit (30%) of the GST system as suggested by recent molecular dynamic calculations^31^,^39^.

**Figure 6:**
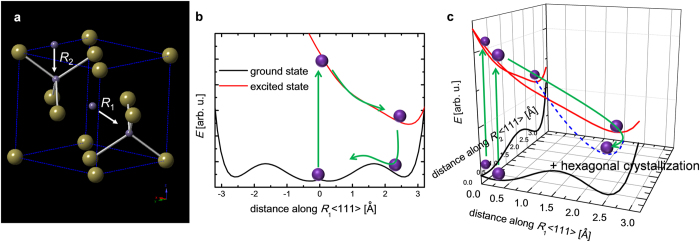
Summary of the amorphization process in GST. (**a**) Local amorphization with disordering of Ge atoms along the <111> directions for representative displacement coordinates (R_1_ and R_2_). (**b**) Amorphization and subsequent recrystallization below the fluence threshold. (**c**) Permanent amorphization via a multi-displacement effect above the fluence threshold. The purple spheres represent germanium atoms. The potential energy surfaces (black lines for the ground state and red lines for the excited state) are calculated from Ref. 41.

The heat generated in GST after the photoexcitation should be considered because the self-reversible amorphization in GST below the fluence threshold and the hexagonal recrystallization above the fluence threshold are involved in thermal processes. Following the energy conservation law, the change in lattice temperature after photoexcitation below the fluence threshold is estimated as


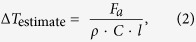


where *F*_*a*_ is the fluence of the absorbed laser (2 mJ cm^–2^), and *ρ*, *C* and *l* are the density (6.35 g cm^–3^), specific heat^40^ (0.205 J g^–1^ K^–1^) and thickness (2 × 10^–6^ cm) of the material. The increase in temperature (Δ*T*_estimate_ = 770 K) estimated by eq. 2 is far above the lattice temperature obtained from the electron diffraction measurement (Δ*T* ≈ 100 K), which is even higher than the melting temperature (600 °C). This mismatch suggests that the photoexcited GST is in an electronically excited state after ~100 ps and/or that most of the energy deposited in the system is used for breaking and reconnecting the Ge–Te bonds^36^. After several hundred picoseconds or a few nanoseconds, the local heat brings the displaced tetrahedral Ge atoms back to the octahedral sites via an annealing process. The simple amorphization process triggered by a Ge-atomic displacement along the [111] direction with the schematic drawing of the ground and excited state potential energy surfaces^41^ in Fig. 6a,b. This result, in combination with the low heat generation present that the amorphization process of GST is related to a laser-induced nonthermal process.

The local displacements of Ge atoms from an octahedral to a tetrahedral arrangement are saturated at approximately 25%; therefore, the dynamics of the permanent amorphization observed above the fluence threshold is more complicated. Because more than two Ge atoms in a unit cell are displaced simultaneously in the over-saturated state, we must define more than two Ge-atomic displacements in the <111> directions (Fig. 6a). The multiple Ge-atomic displacements should be represented by a multi-dimensional potential surface and should have a potential minimum that cannot be defined in one dimension, leading to permanent amorphization (Fig. 6c). Most of the deposited energy should be used for breaking and reconnecting the Ge–Te bonds (*vide supra*); however, these bond-breaking and reconnecting effects should also be saturated in these over-saturated conditions, and the deposited energy should be efficiently transduced to heat. Subsequently, the local lattice temperature should exceed the H-phase transition temperature (Δ*T* > 300 K), creating a fraction of hexagonal crystals. This irreversible hexagonal crystal growth in GST may be one of the possible damage mechanisms, unveiled by near-UV pulsed laser irradiation above the fluence threshold with a crystallographic methodology.

In summary, we observed the fundamental processes of self-reversible and permanent nonthermal amorphization in GST. The photoexcited R-phase GST is electronically stabilized within 1 ps, followed by the displacements of Ge atoms in 20 ps. Our findings support the theory that the laser-induced nonthermal amorphization is initiated from Ge-atomic displacements along the <111> directions from octahedral to tetrahedral sites. Above the threshold corresponding to more than 25% of Ge-atomic displacements, permanent amorphization occurs through a multi-displacement effect inside a unit cell, leading to a fraction of damage core, *i.e.*, irreversible hexagonal crystal growth. Because the dynamics of amorphization in GST is rather complicated, it is also important to observe atomic motions during the amorphization process using ultrabright single-shot x-ray diffraction^42^,^43^ and XANES spectroscopy which will provide precise transient atomic coordinates or optical spectroscopy which will indirectly measure the evolution of the coherent lattice response^44^.

## Methods

### Tr-ED experiments and single-shot optical pump-probe experiments

Tr-ED measurements were performed in transmission mode. The experimental detail are given elsewhere^23^. Pump light at a wavelength of 400 nm was focused to a 280-μm spot size on a polycrystalline GST film (20 nm thick). To avoid any accumulative heat, the repetition rate was limited to 500 Hz. The fluence of the incident laser was 5–10 mJ cm^–2^ (absorption fluence: 2–4 mJ cm^–2^). The acceleration voltage of the probe-pulsed electron was 75 keV generated with a DC electric field. Photoinduced structural changes inside the material were followed with 1-ps pulses containing 2.0 × 10^4^ electrons confined to a 100-μm diameter spot on the GST sample. Diffracted and directly transmitted electrons were focused with a magnetic lens onto a 1:2 fibre-coupled CCD camera (ikon-L HF, Andor ) coated with a P43 (Gd_2_O_2_S:Tb) phosphor scintillator. For each electron diffraction pattern, 5.0 × 10^3^ shots of electron pulses were accumulated.

The experimental details of the single-shot optical pump-probe setup are given in Ref. 12. The output from a Ti:sapphire amplifier system was divided into two beams; one beam was converted to near-UV light (400 nm) by focusing on a BBO crystal and was then used as the pump pulse, whereas the other beam was used to generate a white-light continuum by focusing on a CaF_2_ thin plate. The white-light continuum used as the probe illuminated the echelon mirror, which had a micro-step structure. The probe beam diffracted by the echelon mirror was focused onto a sample, together with the pump pulse. After passing through the sample, the probe beam was collimated and focused on the entrance slit of a monochromator coupled with a two-dimensional CCD camera.

### Calculation of electron diffraction intensity

The electron diffraction intensity was calculated in the framework of the kinematic theory of diffraction using the CrystalMaker®^45^ software. The CrystalMaker® software provides relative x-ray diffraction intensities. Because the main differences between electron diffraction and x-ray diffraction are atomic scattering factors, we used the Laue function and atomic coordinates from CrystalMaker® and applied the values of atomic scattering amplitudes for electrons and neutral atoms given in Ref. 46.

### Sample preparation

For electron diffraction measurements, 20-nm-thick GST films were deposited on 30-nm-thick SiN self-standing membranes using the RF magnetron sputtering method. The GST films were covered with a 10-nm-thick SiO_2_ protecting layer. The as-deposited amorphous GST films were annealed at a temperature of ~430 K for two minutes for the transition to R-phase GST. For time-resolved optical spectroscopy, 10-nm-thick GST films were prepared on SiO_2_ substrates with the same procedure.

## Additional Information

**How to cite this article**: Hada, M. *et al.* Ultrafast time-resolved electron diffraction revealing the nonthermal dynamics of near-UV photoexcitation-induced amorphization in Ge_2_Sb_2_Te_5_. *Sci. Rep.* **5**, 13530; doi: 10.1038/srep13530 (2015).
